# A Methodological Safe-by-Design Approach for the Development of Nanomedicines

**DOI:** 10.3389/fbioe.2020.00258

**Published:** 2020-04-02

**Authors:** Mélanie Schmutz, Olga Borges, Sandra Jesus, Gerrit Borchard, Giuseppe Perale, Manfred Zinn, Ädrienne A. J. A. M Sips, Lya G. Soeteman-Hernandez, Peter Wick, Claudia Som

**Affiliations:** ^1^Technology and Society Laboratory, Empa – Swiss Federal Laboratories for Materials Science and Technology, St. Gallen, Switzerland; ^2^Center for Neuroscience and Cell Biology, Faculty of Pharmacy, University of Coimbra, Coimbra, Portugal; ^3^School of Pharmaceutical Sciences Geneva-Lausanne, Geneva, Switzerland; ^4^Polymer Engineering Laboratory, Department of Innovative Technologies, Mechanical Engineering and Materials Technology Institute, University of Applied Sciences and Arts of Southern Switzerland, Manno, Switzerland; ^5^Institute of Life Technologies, University of Applied Sciences and Arts Western Switzerland (HES-SO Valais-Wallis), Sion, Switzerland; ^6^National Institute for Public Health and the Environment, Bilthoven, Netherlands; ^7^Particles-Biology Interactions Lab, Empa – Swiss Federal Laboratories for Materials Science and Technology, St. Gallen, Switzerland

**Keywords:** Safe-by-Design, polymeric nanobiomaterials, nanocarriers, drug delivery, nanomedicine

## Abstract

Safe-by-Design (SbD) concepts foresee the risk identification and reduction as well as uncertainties regarding human health and environmental safety in early stages of product development. The EU’s NANoREG project and further on the H2020 ProSafe initiative, NanoReg2, and CALIBRATE projects have developed a general SbD approach for nanotechnologies (e.g., paints, textiles, etc.). Based on it, the GoNanoBioMat project elaborated a methodological SbD approach (GoNanoBioMat SbD approach) for nanomedicines with a focus on polymeric nanobiomaterials (NBMs) used for drug delivery. NBMs have various advantages such as the potential to increase drug efficacy and bioavailability. However, the nanoscale brings new challenges to product design, manufacturing, and handling. Nanomedicines are costly and require the combination of knowledge from several fields. In this paper, we present the GoNanoBioMat SbD approach, which allows identifying and addressing the relevant safety aspects to address when developing polymeric NBMs during design, characterization, assessment of human health and environmental risk, manufacturing and handling, and combines the nanoscale and medicine field under one approach. Furthermore, regulatory requirements are integrated into the innovation process.

## Introduction

The concept of Safe-by-Design (SbD) was addressed in the field of nanotechnology because of the continuous uncertainty about the potentially harmful effects of nanomaterials on humans and the environment. Its implementation started with the Dutch NanoNextNL program^1^ and the European NANoREG project and was further developed by the H2020 ProSafe initiative and H2020 NanoReg2 project (Soeteman-Hernandez et al., 2019). Since then, an increasing number of European Union projects focused on SbD for nanomaterials (Lynch, 2017). Even though various concepts of SbD coexist, they share the purpose of assessing safety as early as possible in the innovation process of a nanomaterial or nanoproducts. They aim at reducing adverse effects on human health and the environment by altering nanoproduct design (Soeteman-Hernandez et al., 2019) and by ensuring safety along its lifecycle (Bottero et al., 2017; Kraegeloh et al., 2018). The SbD concept is therefore different from conventional risk assessment approaches, which only consider safety when the product is already fully developed (Schwarz-Plaschg et al., 2017).

Despite being a rather novel concept in the context of nanotechnology, the principle behind SbD is not new and already applied by other industries (Kraegeloh et al., 2018). The medicine field has also long expertise in ensuring safety throughout the drug discovery and development process (Hjorth et al., 2017). However, how to handle safety issues effectively at the very beginning of drug development, to allow the selection of drug candidates, and mitigate toxicity is still being investigated (Kramer et al., 2007; Loiodice et al., 2019). The concept of Quality-by-Design (QbD) is widely used by pharmaceutical industry and its implementation is foreseen by the pharmaceutical development guidelines. The SbD is a new concept for the Pharmaceutical industry and it is not yet included in ICH, EMA, or FDA guidelines. This means that even if safety is considered during the pharmaceutical development, there is no systematic SbD approach yet in place. The concept of QbD presupposes the definition of the critical quality attributes (CQA) that will lead to the achievement of a product with proven effectiveness and the SbD would establish CQA that will lead to a product with high safety.

The application of nanotechnology in the medicine field (nanomedicine) brought new barriers precluding the prediction of potential adverse effects to human health and the environment because of the complexity of nanobiomaterials (NBMs). The unpredictability of nanomedicines’ interaction with biological systems makes it difficult to bring these to the market (Resnik and Tinkle, 2007; Accomasso et al., 2018) and consequently, their potential benefits in medicine are still underexploited (Tinkle et al., 2014; Troiano et al., 2016). The lack of guidelines, standards and tools adapted to nanomedicines for assessing their risks represents one of the causes for this situation (Accomasso et al., 2018).

The development of such products remains therefore challenging. In addition, nanomedicines are costly and based on an interdisciplinary approach. They are at the junction of pharma, medtech, biotech and nanotech companies, and academia, which are important economic and social players in Switzerland and Europe. These companies may have different roles in the value chain of nanomedicines’ development and as they have different backgrounds, they may have various needs to overcome the complexity of nanotechnology for medical applications.

As there is no systematic SbD approach in place for nanomedicines and that not all actors (coming from different fields) are experienced in considering safety to reduce risks on human health and the environment, there is a need for a methodological approach enabling to consider all necessary aspects to evaluate the safety of nanomedicines early during product development. This would ultimately improve the efficiency of the innovation process and the collaboration of all involved interdisciplinary actors and thus ensure the development of a safe product from the beginning of the process.

In order to fill this gap, within the GoNanoBioMat project,^2^ we aimed at elaborating a methodological SbD approach by taking up the principles of the SbD approach developed for nanotechnologies in general and by adapting it to the field of nanomedicines. The developed methodological approach has a focus on polymeric nanocarriers for drug delivery (Som et al., 2019) as they are valuable materials, widely used to prepare nanoparticles and microparticles for the purpose of encapsulating drugs (Etheridge et al., 2013), can be biodegradable, biocompatible, and can be tailored to have targeting abilities (Bennet and Kim, 2014; Moritz and Geszke-Moritz, 2015). Therefore, these materials are expected to increase drug efficacy and safety (Ariën and Stoffels, 2016).

The aim of this paper is (1) to present what we adapted from the SbD concept developed within the EU projects NANoREG and NanoReg2, and the ProSafe initiative (hereafter general SbD approach) for the field of nanomedicines and (2) present the methodological SbD approach (hereafter the GoNanoBioMat SbD approach).

## Adaptation of the General SbD Approach to Nanomedicines

The general SbD approach can be applied in many different fields (e.g., paints, textiles, etc.), is addressed to industries, and can be used by regulators as a reference tool (Kraegeloh et al., 2018). Its goal is to “*reduce uncertainties and risks of human and environmental safety of nanotechnology, starting as early as possible during the innovation process, on the basis of mandatory and voluntary safety and efficacy compliance requirements*” (Soeteman-Hernandez et al., 2019). The main elements of the general SbD approach are: (1) it uses a stage-gate innovation approach, (2) it is based on three pillars, which are *Safe materials and products*, *Safe production*, and *Safe use and end-of-life*; (3) it includes SbD action for maximizing safety while maintaining functionality, and (4) it is integrated into a *Safe Innovation Approach* (see extensive description in Kraegeloh et al., 2018; Soeteman-Hernandez et al., 2019). Below we show how we changed or adapted these elements of the general SbD approach to nanomedicines (the comparison can be seen in Table 1).

**TABLE 1:** Comparison of the general SbD approach developed by NANoREG, NanoReg2, and the ProSafe initiative with the GoNanoBioMat SbD approach.

Comparison of the general and GoNanoBioMat SbD approaches	

*General SbD approach*	*GoNanoBioMat SbD approach*
Built on the stage-gate innovation approach	Built on an iterative approach

Based on three design pillars:	Based on three design pillars:
(1) Safe materials and products for human health and the environment	(1) Safe Nanobiomaterials: designing low-hazard NBMs for specific drug delivery applications by assessing human health and environmental risks
(2) Safe production for occupational health	(2) Safe Production: manufacturing and control of NBMs to ensure their safety and quality
(3) Safe use and end-of-life for preventing exposure during use and having adapted recycling and disposal routes	(3) Safe Storage and Transport: ensuring the safety and quality of NBMs

It includes Safe-by-Design actions for maximizing safety while maintaining functionality	It includes Safe-by-Design actions for maximizing safety while optimizing efficacy and costs

It is integrated into a Safe Innovation Approach (SIA), which combines the SbD concept and the Regulatory Preparedness (RP) concept. It provides a Trusted Environment (TE), which is a space for enabling a dialogue among stakeholders and regulators for sharing and exchanging knowledge on nanomaterials	It is embedded into and frames the guidelines, which provides the state of scientific knowledge by meta-analysis, specific methods for production of nanocarriers, relevant endpoints to test, and safety aspects to consider

As can be seen in Table 1, the GoNanoBioMat SbD approach is not based on a stage-gate innovation approach. Instead, it is an iterative approach. This decision was made in order to better represent the reality of “drug discovery and development” field, which also uses an iterative approach (Hjorth et al., 2017). In addition, the iterations are necessary to build up knowledge, as it will be shown in the section “GoNanoBioMat SbD Approach,” on physico-chemical properties and their biological effects. This is because currently, it is not possible to predict these effects only based on literature and modeling.

The GoNanoBioMat SbD approach is also based on three pillars (Table 1), but these were modified to match the scope of the topic at hand. The pillar *Safe Nanobiomaterials* corresponds to the first pillar of the general SbD approach and has the same aim. In the general and GoNanoBioMat SbD approaches, the second pillar is *Safe Production*. However, the focus in the GoNanoBioMat SbD approach is not only on the safety of workers but also on ensuring safety and quality of the NBMs and on applying good manufacturing practices (GMP), which are a prerequisite to produce medicines and consequently nanomedicines. On the one hand, the third pillar of the general SbD approach is about *Safe use and end-of-life*. Its main goal is to prevent exposure during use and to have adapted recycling and disposal routes. On the other hand, the third pillar of the GoNanoBioMat SbD approach is about *Safe Storage and Transport* in order to ensure the safety and quality of NBMs because they may experience transformations (Cobaleda-Siles et al., 2017), which may affect their safety and quality (USP36–NF31,2012; European Commission, 2013). Storage, and more particularly shelf-life, is an aspect being highly connected to the logistics and costs of the final nanomedicine and therefore its viability on the market (Diven et al., 2015). As can be seen, here the pillar is a bit narrower than in the general approach. This is to better represent the needs for developing nanomedicines.

Both approaches include SbD actions (Table 1). The difference between the two is that the functionality is specified into efficacy in the GoNanoBioMat SbD approach. It was changed into efficacy because efficacy is a measurement of the successful pharmacological effect of a drug and therefore more representative for developing nanomedicines. The goal of these SbD actions is to maximize safety while optimizing efficacy and costs by comparing different forms of NBMs. However, it should be pointed out that sometimes it is not feasible to maximize both efficacy and safety at the same time (Soeteman-Hernandez et al., 2019). Optimization will always require iterations in order to be able to balance efficacy and safety (Hjorth et al., 2017).

Finally, in Table 1 it is possible to see that the general SbD approach is integrated into a *Safe Innovation Approach* and provides a *Trusted Environment* (Soeteman-Hernandez et al., 2019). The *Safe Innovation Approach* combines the SbD concept and the *Regulatory Preparedness* concept. The *Regulatory Preparedness* concept being the improvement of anticipation of regulators to keep up with the fast growing knowledge on nanomaterials and thus facilitate the development of adaptable regulations. The *Trusted Environment* is a space for enabling a dialogue among stakeholders and regulators for sharing and exchanging knowledge on nanomaterials. The GoNanoBioMat SbD approach, however, is embedded into and sets the frame for a document whose title is “Guidelines for implementing a SbD approach for medicinal polymeric nanocarriers” written and published by the GoNanoBioMat project consortium (Som et al., 2019). The guidelines provide the state of scientific knowledge with meta-analyses, decision trees, methods for producing NBMs, relevant endpoints to test, and safety aspects to consider early and throughout the development of polymeric NBMs for drug delivery. The guidelines can be downloaded under this link: www.empa.ch/gonanobiomat.

## GoNanoBioMat SbD Approach

**FIGURE 1:**
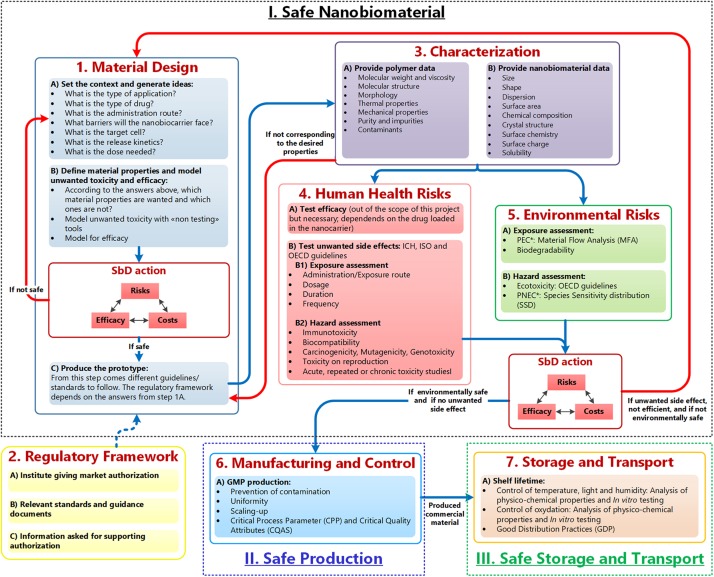
GoNanoBioMat SbD approach. The blue arrows correspond to the flow of polymeric NBMs from design to storage and transport. The red arrows are feedback loops used whenever the NBM is unsafe, or inefficient. Adapted from Som et al. (2019). *PEC, predicted environmental concentration; PNEC, predicted no effect concentration.

As mentioned, the GoNanoBioMat SbD approach is a methodological approach for developing nanomedicines with a focus on polymeric NBMs for drug delivery and is presented in Figure 1. It contains the following steps: *Material Design*, *Characterization*, *Human Health* and *Environmental Risks* (first pillar), *Manufacturing and Control* (second pillar), and *Storage and Transport* (third pillar). The regulatory framework for developing nanomedicines is also included within the approach starting at the end of the *Material Design* step. The bullet points inside the boxes correspond to methods and tools that can be used or endpoints that should be considered and tested in each step. The blue arrows represent the flow of polymeric NBMs from their design until their storage and transport. The red arrows are feedback loops (iterations) going back to the *Material Design* step.

It is important to note, that most of these steps also apply to other NBMs and other type of nanomedicine applications. For example, the *Human Health* and *Environmental Risks* steps could be applied to any type of NBMs. However, in the *Material Design* step and in the *Characterization* step, specific questions (e.g., what is the type of drug and what is the release kinetics) for drug delivery and specific parameters to characterize polymers are provided, respectively. Therefore, the total of questions only applies to polymeric NBMs for drug delivery, even if many questions also apply to other NBMs or other applications.

As can be seen in Figure 1, the GoNanoBioMat SbD approach starts with the *Material Design* step. This step is divided into three sub-steps, which are (a) set the context and generate ideas, (b) define material properties and screen for unwanted toxicity and efficacy, and (c) produce the prototype.

In the first sub-step, a set of questions can be used to guide the conceptual process for developing NBMs for drug delivery, and searching for the relevant literature. The questions include the type of application, type of drug (possibility of chemical interaction between drug and polymer), administration route, the biological barriers, target cells, release kinetics, and dose needed. All these aspects influence the design of nanocarriers (Elsabahy and Wooley, 2012), in other words, its physicochemical properties to be efficient as a drug delivery system and lining up for safe application. An important consideration to bear in mind is that the properties of the polymer (particles larger than 1 micron) may not be equal to the properties of the polymer when the size of its particles is reduced to the nanoscale. Once the data from literature are collected, the data can be used to screen for efficacy but also toxicity and to define the wished material properties of the nanocarriers (second sub-step) by using modeling tools (i.e., non-testing tools), such as quantitative structure–activity relationship tools (OECD, 2007). These tools have for aim to find a correlation between NBMs properties and their corresponding effect (e.g., cell internalization, cytotoxicity) and may enable to assess whether a material is safe for medical purposes. However, it has to be noted that such methods still need to be further developed.

As aspects of safety and functionality should be taken into account at the very beginning of the project’s conception (Cobaleda-Siles et al., 2017), these two sub-steps based on literature and modeling are facilitating their consideration. However, assessing the human health risks in an early stage of innovation only based on data found in the literature is currently not adequate. This may be a result of the lack of standardized assays, which lead to a high variation in reported studies (Hofmann-Amtenbrink et al., 2015). Also some studies have no proper characterization and lack appropriate controls specific to the nanoscale (Jesus et al., 2019), which makes comparisons between toxicity outcomes difficult. Therefore, experimental studies are still needed.

After these two sub-steps, comes the first SbD action. Its goal is to compare different possible NBMs for the intended use/application, which was defined in the beginning of the *Material Design* step, and to select the NBMs having a good balance between, safety, efficacy, and costs. After this, the selected NBMs should be produced as prototypes.

These prototypes should be then characterized in order to be able to find relationships between physicochemical properties of NBMs and their biological effects, and thus apply the concept of SbD. As can be seen in Figure 1, the properties attributed to the polymer itself (e.g., molecular weight) and the properties attributed to the nanosize (e.g., size) should be characterized. If the desired properties of the prototypes do not correspond to the measured properties, the prototypes should go back to the prototype production sub-step in order to optimize the production process. One criterion in SbD requires understanding the variables contributing to undesired side effects (Lin et al., 2018). Therefore, to have a thorough characterization of polymeric NBMs, the *Characterization* step includes specific parameter to be tested for polymers NMBs, such as molecular weight, size and surface area. This step is also essential to determine later the CQAs, which are defined as “physical, chemical, biological, or microbiological properties or characteristics that should be within an appropriate limit, range, or distribution to ensure the desired product quality” (ICH Q8 (R2), 2009).

The next two steps are experimental steps to evaluate the human health and the environmental risks of the selected NBMs. For both, the exposure and the hazard should be evaluated. For the *Human Health Risks* step, the route of administration/exposure, the dosage, the duration and frequency should be determined as safety of NBMs depends on the route of administration/exposure and the resulting respective pharmacokinetic profiles (Jesus et al., 2019). For the hazard, the following endpoints should be tested: immunotoxicity, biocompatibility, carcinogenicity, mutagenicity, genotoxicity, toxicity on reproduction, acute, repeated, or chronic toxicity studies. All tested endpoints should as well include appropriate controls for the nanoscale. The proposed endpoints are in line with current regulation (Jesus et al., 2019).

In parallel, the assessment of the environmental risks should be performed. To do so, the predicted environmental concentration and the predicted no effect concentration have to be calculated (Hauser et al., 2019). The former can be assessed via a material flow analysis and the latter via performing a (probabilistic) species sensitivity distribution. For this, ecotoxicity data are needed, which can be obtained either via literature or experimentally by following OECD guidelines.

After the *Human Health* and *Environmental risks* steps comes the second SbD action. As for the first one, the goal is to compare the selected NBMs and choose the one maximizing safety, while optimizing efficacy and costs. At this point, either one NBM is selected as the final candidate or if no NBMs have a good balance between benefits and risks, the developer should go back to the *Material Design* step. The results of these two steps can help to build up a useful database. In other words, with iterations, a database with the experimental results could be established and these data could be used for modeling. Ultimately, it would enable better predictions of NMBs’ efficacy and toxicity.

If one final candidate has been selected, the developer of NBMs should go to the *Manufacturing and Control* step. The goal of this step is to scale-up the production by applying GMP, preventing contamination and ensuring uniformity between the batches. In this step, CQAs of NBMs must be identified as well as Critical Process Parameters. These are defined as the “process parameters that influence CQAs and therefore should be monitored or controlled to ensure the process produces the desired quality” (ICH Q8 (R2), 2009). It can be noted that this step is typically valid for any type of NBMs.

After scale-up, usually the nanocarrier and their encapsulated drug system would go to clinical trials. However, as we did not include clinical trials in the approach because it was out of the scope of the project, the next step is *Storage and Transport*. The (nano)medicine stability studies have to be performed (SME Office, 2016; MDR, 2017), because nanocarriers and encapsulated drug, both, or just one of them, might experience degradation process during their life cycle, which might affect the quality and safety of the nanomedicine (Cobaleda-Siles et al., 2017).

Finally, the Swiss and European regulatory frameworks for the marketing authorization of nanomedicine is embedded within the GoNanoBioMat SbD approach. More information on this aspect can be directly found in the GoNanoBioMat guidelines.^3^

## Discussion

In case of nanomedicines, SbD approaches should be included in the International Council for Harmonisation of Technical Requirements for Pharmaceuticals for Human Use (ICH) guidelines and relevant OECD guidance and guidelines. ICH is unique in bringing together the regulatory authorities and pharmaceutical industry to discuss scientific and technical aspects of drug registration and thus to discuss what should be included within guidelines concerning the safety of the NBMs and nanomedicines.

The GoNanoBioMat SbD approach is methodological, contains all important elements to consider in order to integrate safety early and throughout the development of polymeric NBMs for drug delivery. It can as well to a certain extent be applied to other types of NBMS and nanomedicine applications. Including safety in the design of NBMs is an important aspect, especially for nanomedicines, which are highly regulated, cost and time consuming, and complex. However, the approach should not be seen as a warranty of complete safety, because absolute safety is unreachable (Cobaleda-Siles et al., 2017; Hjorth et al., 2017; van de Poel and Robaey, 2017), and should therefore be considered as a design strategy (van de Poel and Robaey, 2017) since the past showed that each nanomedicine has to be taken as case-by-case (McNeil, 2009). As for the general SbD concept, it has no legal binding and does not replace regulatory requirements (Soeteman-Hernandez et al., 2019).

The GoNanoBioMat SbD approach was focusing only on the safety of nanocarriers (polymeric NBMs) and not on the nanocarriers and its encapsulated drug. For regulatory purpose, it is necessary to test the safety of the nanocarrier alone in addition to the nanocarrier/drug system. Therefore, this GoNanoBioMat SbD approach is a first step toward the integration of safety early in the development of such products. Efficacy, which is closely related to the drug used, could not be included in the approach and therefore must be evaluated case-by-case. In a future development of the GoNanoBioMat SbD approach adding steps for clinical trials and use will be developed.

Finally, we believe that the GoNanoBioMat SbD approach as presented here may facilitate the implementation of the general SbD concept and to find a balance between benefits and risks by comparing different nanocarrier candidates in terms of their respective safety, efficacy, and costs. For instance, the GoNanoBioMat SbD approach provides all relevant steps for developing polymeric NBMs; provides methodology and endpoints to test human health and environmental risks, which are in line with current regulations; is an iterative process; and combines the nanoscale and medicine field under one methodological approach. In addition, the approach may bring the different actors of the value chain on a common ground. Ultimately, the approach may enable to move toward safe and efficient NBMs, safe production, and safe storage and transport.

## Author COntributions

MS created the GoNanoBioMat SbD approach in collaboration with CS, OB, SJ, PW, GB, GP, ÄS, and LS-H. MS wrote the manuscript. CS wrote some part of the manuscript. OB, SJ, PW, GB, GP, ÄS, and LH read and reviewed the manuscript. All authors approved the submitted version.

## Conflict of Interest

The authors declare that the research was conducted in the absence of any commercial or financial relationships that could be construed as a potential conflict of interest.

## Footnotes

1 www.nanonextnl.nl

2 The GoNanoBioMat project was initiated by the ProSafe initiative

3 www.empa.ch/gonanobiomat
